# Folic Acid-Modified Ibrutinib-Loaded Silk Fibroin Nanoparticles for Cancer Cell Therapy with Over-Expressed Folate Receptor

**DOI:** 10.3390/pharmaceutics15041186

**Published:** 2023-04-07

**Authors:** Marta G. Fuster, Mercedes G. Montalbán, Imane Moulefera, Gloria Víllora, David L. Kaplan

**Affiliations:** 1 Department of Chemical Engineering, Faculty of Chemistry, University of Murcia (UMU), Campus de Espinardo, 30100 Murcia, Spain; 2 Department of Biomedical Engineering, Tufts University, Medford, MA 02155, USA

**Keywords:** silk fibroin, folate receptor, ibrutinib, nanoparticles, cancer, HeLa, BT-474, SKBR3, EA.hy926

## Abstract

The anticancer drug ibrutinib (IB), also known as PCI-32765, is a compound that irreversibly inhibits Bruton’s tyrosine kinase (BTK) and was initially developed as a treatment option for B-cell lineage neoplasms. Its action is not limited to B-cells, as it is expressed in all hematopoietic lineages and plays a crucial role in the tumor microenvironment. However, clinical trials with the drug have resulted in conflicting outcomes against solid tumors. In this study, folic acid-conjugated silk nanoparticles were used for the targeted delivery of IB to the cancer cell lines HeLa, BT-474, and SKBR3 by exploiting the overexpression of folate receptors on their surfaces. The results were compared with those of control healthy cells (EA.hy926). Cellular uptake studies confirmed total internalization of the nanoparticles functionalized by this procedure in the cancer cells after 24 h, compared to nanoparticles not functionalized with folic acid, suggesting that cellular uptake was mediated by folate receptors overexpressed in the cancer cells. The results indicate that the developed nanocarrier can be used for drug targeting applications by enhancing IB uptake in cancer cells with folate receptor overexpression.

## 1. Introduction

The fight against cancer is one of the greatest medical challenges in the history of mankind and the second-leading cause of death worldwide [1], with almost 10 million deaths in 2020. According to the World Health Organization (WHO), the most common types of cancer are breast, lung, colon, rectum, and prostate cancer. Alongside radiotherapy and surgery, chemotherapy is one of the most widely used treatments; however, the use of chemotherapeutic drugs is limited by their cytotoxicity, low stability, and acute side effects [2]. These drugs circulate throughout the body, reaching the area of therapeutic action but also negatively impacting healthy cells, which are often severely damaged or die. To reduce side effects, enhancing the efficiency of drugs and their delivery to targeted sites using safe and effective drug delivery systems is a top priority [3].

Advancements in nanotechnology have had a significant impact on cancer treatments, especially drug delivery systems. Modification of the physiochemical properties of nanomaterials such as nanoparticles can improve control over the biodistribution, pharmacokinetics, and pharmacodynamics of loaded drugs compared to free drugs [2]. Most drugs involved in cancer treatments are poorly water-soluble or hydrophobic, so they are quickly eliminated by the organism. Nanoparticles can enhance the solubility and the stability of these drugs, promote transport across membranes, prolong circulation times, and facilitate their accumulation in the tumor rather than impacting healthy tissues [4].

Significant research focused on the synthesis of biopolymeric nanoparticles as drug delivery vehicles, including chitosan, albumin, alginate, gelatin, and silk fibroin, due to their biocompatibility, biodegradability, and low immunogenicity [5,6,7]. Among these natural polymers, silk fibroin (hereafter referred to as silk), mostly extracted from the cocoons of domesticated silkworms (*Bombyx mori*), is an interesting biomaterial for medical applications [8]. Silk is an FDA-approved polymer that has been popularly used in numerous medical applications such as sutures, tissue regeneration, coating devices, and drug delivery systems for cisplatin [9], doxorubicin [10], paclitaxel [11], curcumin [12,13], naringenin [14], or rosmarinic acid [15], among others. During the last few years, silk has gained increasing interest due to its excellent mechanical properties and high biocompatibility, biodegradability, inexpensiveness, and preparation flexibility. These characteristics are ideal for the synthesis of silk nanoparticles. Several factors, such as the molecular weight and crystallinity of the silk, the encapsulated drug properties, and the synthesis conditions influence the properties of the particle (mean size, size distribution, surface zeta potential, drug loading and release profiles, or stability). Organic solvents play an important role in the synthesis of the silk fibroin nanoparticles. Methanol or ethanol are good antisolvents to obtain silk nanoparticles from silk fibroin aqueous solution, whereas acetonitrile only gives aggregates without forming nanoparticles [16].

Previous studies have shown the feasibility of the silk nanoparticles being administered by parenteral [17], transdermal [18], oral [19], ocular [20], and orthopedic [21] routes.

In order to avoid the indiscriminate action of antitumor drugs, the functionalization of the surface of silk nanoparticles with specific ligands is a logical approach for targeted treatment. Tumor tissues overexpress membrane receptors, and among them, the folate receptor has been of interest because tumor cells consume large quantities of folate [22]. Hence, the folate receptor is overexpressed in the majority of tumor cells, whereas its expression is limited in healthy cells [22]. For this reason, folic acid is a suitable molecule to display for active targeting in cancer treatments as an essential cofactor in the synthesis of purines and pyrimidines and other cellular methylation reactions, including DNA, proteins, and lipids [23]. Folic acid retains a high affinity for folate receptor alpha via its g-carboxylic acid group. Folic acid has been used for the targeted delivery of drug loaded nanoparticles. For example, the encapsulation of baicalin in folic acid-modified albumin nanoparticles improved the antiproliferative activity of the drug in human breast cancer cells (MCF-7) by inducing autophagy and apoptosis [24]. Doxorubicin loaded into folate-conjugated magnetic nanoparticles increased cellular uptake, enhanced drug accumulation, and suppressed tumor growth in breast cancer models [25]. Folate-functionalized chitosan nanoparticles improved the anticancer efficiency of cytarabine in MCF-7 cells [26]. However, studies comparing the cytotoxic activity of the cancer drug on tumors and healthy cells remain limited.

In the case of silk nanoparticles, their functionalization with folic acid for targeted drug delivery has not been studied in depth. Silk fibroin-folate nanoparticles were loaded with doxorubicin and evaluated for cytotoxicity on a human breast adenocarcinoma cell line (MDA-MB-231) [27]. Later, the efficiency of folate-conjugated silk nanoparticles loaded with the anticancer model drug doxorubicin was studied with a silk-based 3D matrix using osteoblast-like cells (MG63) and human adenocarcinoma (MDA-MB-231) cells, where the drug-loaded folate-conjugated nanoparticles targeted the cancer cells in terms of 3D bone metastasis in vitro [22]. Doxorubicin was delivered in folate-conjugated silk particles and evaluated in vitro on two cancer cell lines, Hela and raw 264.7, although comparisons with healthy cell controls were not included [28].

In the present work, ibrutinib was used as a drug to inhibit the proliferation of several types of cancer cells. Ibrutinib (PCI-32765) is an orally administered, potent, irreversible inhibitor of Bruton’s tyrosine kinase (BTK) [29]. BTK plays a key role in cell proliferation and cell survival and is a mediator of proinflammatory signals [30]. BTK belongs to a group of 11 tyrosine kinases, including EGFR, ErbB2, ErbB4, Jak3, and BLK [31]. Specifically, Cys797 in EGFR, Cys805 in ErbB2 (HER2), and Cys803 in ErbB4 (HER4) have a homologous active site cysteine sensitive to ibrutinib [32]. Ibrutinib inhibited the phosphorylation of EGFR, HER2, and HER3 due to a reduction in cell viability in HER2 breast cancer cell lines [33]. The overexpression of members of the ErbB family tyrosine kinase was reported in many cancers, and this has proven important as a therapeutic target [34]. Since November 2013, ibrutinib has been approved by the FDA, marketed under the name Imbruvica (Pharmacyclics Inc., Sunnyvale, CA, USA), and has been successfully used for second-line treatment of mantle cell lymphoma. Even these positive capabilities of ibrutinib are associated with significant side effects, such as cutaneous manifestations in 2–27% of patients treated, hair and nail toxicities in 26% and 66% of the patients, respectively, and approximately a 50% risk of bleeding [35].

Therefore, in order to reduce these harmful side effects caused by ibrutinib due to its non-specificity, in this work we functionalized ibrutinib-loaded silk nanoparticles (IB-SFNs) with folic acid (FA). The folic acid-ibrutinib-loaded silk nanoparticles were physio-chemically characterized, and their effectiveness was then studied on HeLa cells (EGFR overexpressed [36]), BT-474 cells (HER2 overexpressed), and SKBR3 cells (HER2 overexpressed [37]). These results were compared with EA.hy926 (healthy cells). To the best of our knowledge, this is the first time that ibrutinib has been used as a target drug using silk nanoparticles as nanocarriers.

## 2. Materials and Methods

### 2.1. Materials

Silk from *Bombyx mori* was sourced from the cocoons of sericulture silkworms maintained in the facilities of IMIDA (Murcia, Spain) and fed on fresh *Morus alba* L. leaves. The cocoons were treated to remove the sericin by boiling in an aqueous solution of Na_2_CO_3_ (0.05 N) twice for 60 min. After washing with pure water and air-drying, the resulting substance had a bright white, cotton-like appearance. The silk was dissolved in the ionic liquid 1-ethyl-3-methylimidazolium acetate [emim+][acetate−] by high-power ultrasound, as described in a previous report [12]. The ionic liquid (97% purity) was obtained from Io-LiTec GmbH (Frankfurt, Germany) and was applied without any further purification. Ibrutinib (IB) [38] (Figure S1 of Supporting Information) was provided by AdooQ Bioscience (Irvine, CA, USA). Highly purified water (18.2 MΩ·cm at 25 °C; from a Millipore Direct-Q1 ultrapure water system, Billerica, MA, USA) was used throughout. N-Hydroxysuccinimide (NHS) and N-(3-Dimethylaminopropyl)-N′-ethylcarbodiimide hydrochloride (EDC) were purchased from Merck. All other chemicals and solvents were of analytical grade and were used without any extra purification.

### 2.2. Preparation of Silk Fibroin Nanoparticles (SFNs)

The preparation of SFNs was based on the method of Montalbán et al. [12] with modifications. Briefly, a silk ionic liquid solution (10% by weight) was prepared by combining 0.5 g silk with 4.5 g [emim^+^][CH_3_COO^−^] using the 3/8″ conical horn of a Branson 450D sonifier (Emmerson Ultrasonic Corporation, Dansbury, CT, USA), at a temperature below 90 °C, and through the use of pulsed ultrasound steps. To lessen viscosity, 3 mL of ultrapure water was slowly added. The mixture was then pumped at 60 °C and sprayed over 100 mL of methanol that was gently stirred at −20 °C with a 0.7 mm two-fluid nozzle thermostatically regulated with compressed N_2_. The nanoparticle suspension was centrifuged in a few steps at 13,400 rpm for 15 min at 4 °C (Sigma 3–18 K centrifuge with a 19,776 H-angle rotor), and after being washed with methanol and water, the SFNs were freeze-dried in an Edwards Modulyo 4K freeze-dryer (Thermo Scientific, Waltham, MA, USA) for 72 h at −55 °C and 0.5 mbar to obtain dry SFNs.

### 2.3. Functionalizing SFNs with Folic Acid (FA-SFNs)

Different methods were utilized to functionalize SFNs with FA.

**Figure 1:**
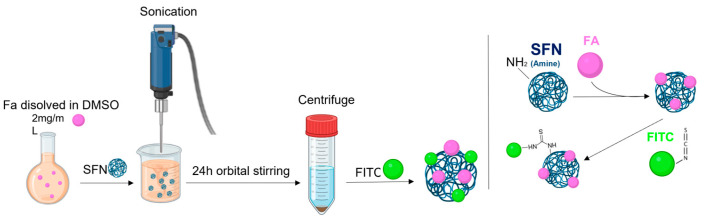
Scheme for the preparation of FA-SFNs by physical adsorption.

**Method 1**: FA-SFNs were obtained by physical adsorption [15]. Briefly, 2 mg/mL of FA was dissolved in DMSO, then 8.5 mg of SFN was added, and sonication was performed for 3 min (amplitude of 30%, pulses of 15 s ON and 15 s OFF). After 24 h of orbital stirring, the samples were centrifuged and washed with deionized water. Then, FA-SFNs (Figure 1) were labeled with fluorescein-5-isothiocyanate (FITC) as described in Section 2.5.

**Figure 2:**
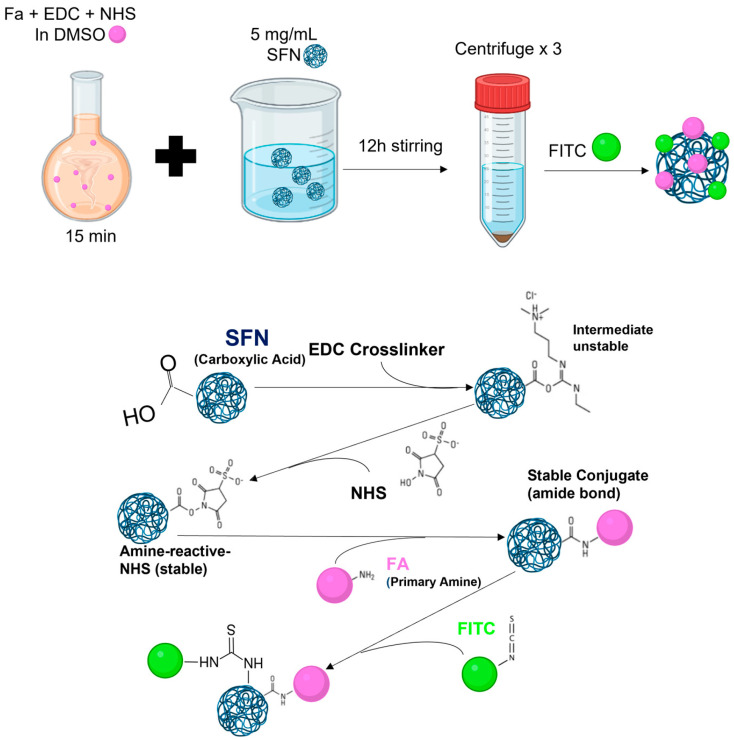
Scheme for the preparation of FA-SFNs by conjugation.

**Method 2**: FA-SFNs were obtained by conjugation with modifications [39]. FA (226.8 mg) was dissolved in DMSO together with EDC and NHS (molar ratio 1:1.5:1.5) for 15 min. Then, the solution was added to 5 mg/mL of SFNs dispersed in water and left to stir overnight. After that, the sample was centrifuged and washed three times with deionized water. Then, FA-SFNs (Figure 2) were labeled with FITC as described in Section 2.5.

**Figure 3:**
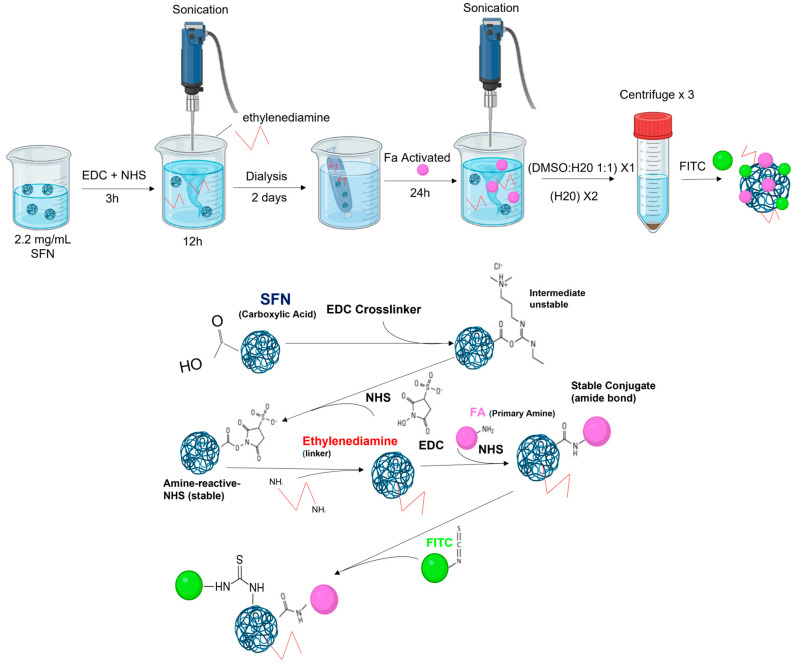
Scheme for the preparation of FA-SFNs by using ethylenediamine as a linker.

**Method 3**: FA-SFNs were obtained with ethylenediamine as a linker [40]. Briefly, 2.2 mg/mL of SFNs was dispersed in water by sonication, as described earlier. Then, EDC (25 mg) and NHS (25 mg) were added at pH 6 under continuous stirring and left to react for 3 h to activate the carboxyl group of SF. Then 1 mL of ethylenediamine was added dropwise into the solution and reacted for 12 h at room temperature with continuous stirring and sonication (amplitude 30%, 1 s ON, 29 s OFF). The reaction mixture was dialyzed against deionized water for 2 days. In addition, 40 mg of FA was dissolved in a 40 mL DMSO/H_2_O (1:1) mixture and activated using EDC (26 mg) and NHS (15 mg) under continuous stirring and sonication (30% amplitude, 1 s ON, 29 s OFF) for 3 h to activate carboxyl groups of FA. The activated FA was added dropwise to the ethylenediamine-SFN conjugate and left to react for 24 h under continuous stirring and sonication (30% amplitude, 1 s ON, 29 s OFF). Then, 3 washes were performed following the protocol:1 wash of DMSO:H_2_O 1:1, 2 washes of H_2_O. Then, the FA-SFNs (Figure 3) were labeled as described in Section 2.5.

**Figure 4:**
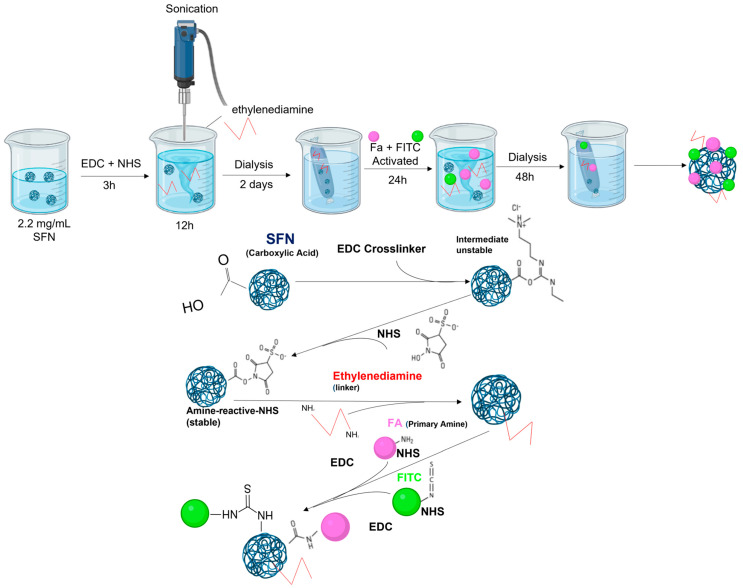
Scheme for the preparation of FA-SFNs using ethylenediamine as a linker and simultaneous labeling and functionalization of ethylenediamine-SFN with FA.

**Method 4**: This method is a variant of Method 3 described above and also uses ethylenediamine as a linker. The two procedures consist of the same steps as dialysis. At this point, labeling and functionalization with FA of ethylenediamine-SFN were carried out at the same time (Figure 4). Here, 10 mg of FITC was dissolved in 50 mL of Milli-Q water, and then EDC (6 mg) and NHS (4 mg) were added at pH 6 with stirring and sonication (30% amplitude, 1 s ON, 29 s OFF) for 3 h. Then, 375 μL of the solution was added dropwise to the dialyzed ethylenediamine-SFNs and the activated FA (as previously described in Method 3) for 24 h of stirring. Then, the mixture was dialyzed against deionized water for 2 days.

**Figure 5:**
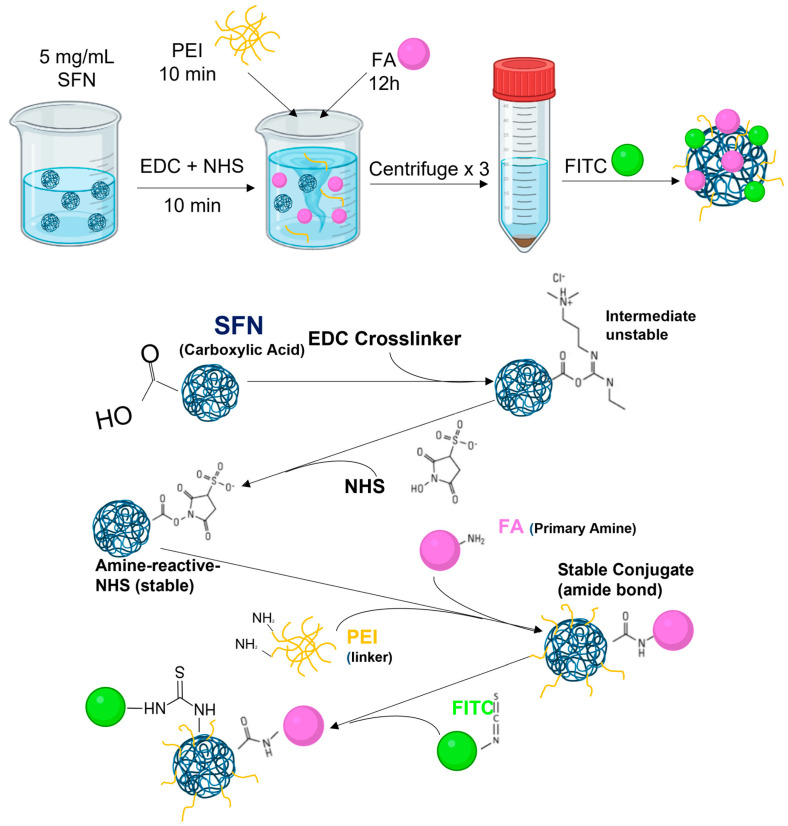
Scheme for the preparation of FA-SFNs using polyethyleneimine as a linker.

**Method 5**: FA-SFNs were attained using polyethyleneimine as a linker [28]. In brief, 100 mg of SFNs were dispersed in 20 mL of ultrapure water. To activate the carboxyl groups of the silk, 15 mg of EDC and 25 mg of NHS were added to the solution and stirred for 10 min. Thereafter, 100 mg of poly(ethyleneimine) (PEI) was added to the solution with stirring for 10 min, and then 100 mg of FA was added. After stirring for 12 h, the mixture was centrifuged and washed with water three times to remove any leftover reagents. Then, FA-SFNs (Figure 5) were labeled as described in Section 2.5.

All methods were carried out at room temperature in the dark.

### 2.4. Formulation of Ibrutinib-Loaded Silk Fibroin Nanoparticles (IB-SFNs/IB-FA-SFNs)

For loading ibrutinib, 20 mL of a 7.5 mg/mL solution of ibrutinib in ethanol were used to resuspend 100 mg of SFNs obtained as described before. The suspension was ultrasonicated for 5 min using 30% amplitude with 15 s ON and 15 s OFF. The dispersion was incubated for 12 h with orbital shaking at 4 °C in the dark. Subsequently, the sample was centrifuged and redispersed by sonication for 3 min at an amplitude of 30% with pulses of 5 s ON and 10 s OFF. It was then examined by Attenuated Total Reflectance Fourier Transformed Infrared Spectroscopy (ATR-FTIR, iS5-Nicolet equipped with an iD7 ATR module, Thermo Fischer Scientific, Waltham, MA, USA) to evaluate the loading of IB on the particles. Finally, the rest of the dispersion was frozen for lyophilization.

The same procedure was used to load ibrutinib onto folic acid-functionalized silk nanoparticles (FA-SFNs).

### 2.5. Labeling Silk Fibroin Nanoparticles with FITC (FITC-SFNs)

**Figure 6:**
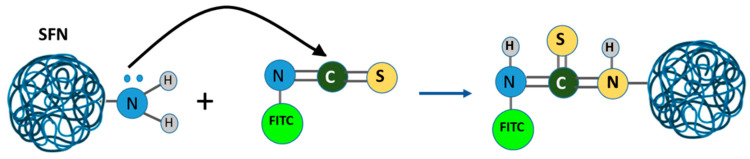
FITC-functionalized protein reaction.

A prior process was used to label SFNs with FITC (Figure 6) [41]. Briefly, a 0.1 M 9.4 pH carbonate-bicarbonate buffer was prepared, and 15 mg/mL of SFNs and 1 mg/mL of FITC were dissolved (1:1 molar ratio) for 2 h with shaking and in the dark. Then, the sample was centrifuged (13,400 rpm, 30 min), and washings were carried out with ethanol-water at 30% *v*/*v* until the supernatant did not show any residual fluorescence.

The same procedure was followed to label the functionalized nanoparticles by Methods 1–5 described above, IB-SFNs (FITC-IB-SFNs) and FA-IB-SFNs (FITC-FA-IB-SFNs).

### 2.6. Physical Characterization of the Nanoparticles

#### 2.6.1. Dynamic Light Scattering (DLS)

The Malvern Zetasizer Nano ZSP, a device from Malvern Instruments Ltd. in Malvern, UK, was utilized to measure the average hydrodynamic diameter (Z-average) and the Z-potential of SFNs, IB-SFNs, and FA-IB-SFNs by using dynamic light scattering (DLS) and phase analysis light scattering (PALS) techniques. The samples were prepared by sonication in ultra-pure miliQ water for 3 min at an amplitude of 30% and pulses of 5 s ON and 5 s OFF using a Branson sonifier 450D. The integrated Malvern software calculated the Z-average and size distribution from the time autocorrelation function of the scattering intensity fluctuation by cumulative analysis. The electrophoretic mobility and Z-potential results shown are the averages of six measurements taken in automated mode by the software with a minimum of 12 runs.

#### 2.6.2. Drug Loading and Encapsulation Efficiency by Attenuated Total Reflectance Fourier-Transform Infrared (ATR-FTIR) Spectroscopy

To determine the drug loading content (DLC) and encapsulation efficiency (EE), a predefined procedure was used. Calibration samples were prepared with a known amount of SFNs and IB, using a 15 mg/mL SFNs dispersion in water and a 5 mg/mL solution of IB in ethanol. Ethanol was added to each sample to maintain a ratio of 30% ethanol to 70% water, which preserved the dissolution of IB and ensured proper dispersion of SFNs. The samples were sonicated for 10 s at an amplitude of 10% to ensure uniformity before measuring their spectra using ATR-FTIR. DLC was derived from the calibration line using Equation (1), while EE was calculated from DLC and the initial mass of IB in the loading solution by mass balance, as expressed in Equation (2).

(1) $$\mathrm{DLC}\;\left(\%\right)=\frac{\mathrm{Mass}\;\mathrm{of}\;\mathrm{IB}\;\mathrm{loaded}\;\mathrm{onto}\;\mathrm{SFNs}}{\mathrm{Mass}\;\mathrm{of}\;\mathrm{IB}-\mathrm{SFNs}}\times 100$$

(2) $$\mathrm{EE}\;\left(\%\right)=\frac{\mathrm{Mass}\;\mathrm{of}\;\mathrm{IB}\;\mathrm{loaded}\;\mathrm{onto}\;\mathrm{SFNs}}{\mathrm{Mass}\;\mathrm{of}\;\mathrm{IB}\;\mathrm{added}\;\mathrm{to}\;\mathrm{SFNs}}\times 100$$

#### 2.6.3. Field Emission Scanning Electron Microscopy (FESEM)

The morphological characterization of the nanoparticles was performed by FESEM using a FEI SciosTM field emission scanning electron microscope (Thermo Scientific, Waltham, MA, USA) operated at 20 kV. Samples of 5 μg/mL were dispersed in water by sonication; a drop was placed on a pedestal and dried by infrared; and then it was coated with platinum.

### 2.7. In Vitro Characterization of Nanoparticles

#### 2.7.1. Cell Culture

Human cervical cancer cells (HeLa), human breast carcinoma cells (BT474 and SKBR3), and human umbilical cord immortalized cells (EA.hy926) were purchased from the American Type Culture Collection (ATCC, Manassas, VA, USA), validated, and stored according to the supplier’s guidelines. Cells were kept in Dulbecco’s Modified Eagle Medium enhanced with 10% (*v*/*v*) thermally deactivated fetal bovine serum, 1 mM glutamax, 1 mM pyruvate, and 1% penicillin-streptomycin. Cells were cultured in a humidified environment with 5% CO_2_ at 37 °C and cultivated in 75 cm^2^ culture flasks. After some passages, the cells were seeded for the tests, and when they achieved confluence, they were removed using a solution of 0.25% trypsin-EDTA.

#### 2.7.2. Inverted Fluorescence Microscopy

To test the binding of FITC and FA to SFNs, nanoparticle samples were brought into contact with live cells in cell culture plates. HeLa and EA.hy926 cell lines were seeded in 6-well plates at a ratio of 70,000 cells/mL culture medium and incubated at 37 °C with 5% CO_2_ for 24 h. The medium was then removed, and a dispersion of the FITC-functionalized (from “Method 5” previously described) and non-functionalized SFNs was added at a concentration of 0.5 mg/mL. The cells were exposed to the nanoparticles for 3 and 6 h, and then photographs were taken at 10× magnification with an inverted fluorescence microscope.

#### 2.7.3. Cell Treatment

The four cell lines HeLa, BT-474, SKBR3, and EA.hy926 were seeded at a density of 15,600 cells/cm^2^. After 24 h, the samples were then fed a medium that had various concentrations of SFNs, FITC-SFNs, IB, IB-SFNs, and FA-IB-SFNs added. The DLC results of IB-SFNs were considered to test the loaded nanoparticles, and a concentration range of 3 × 10^−5^ to 1 mg/mL was assessed in wells using the same concentration of IB in the IB-SFNs as in the experiments involving free IB. The silk-only control was at 3.5 mg/mL to see if the cell viability was affected by the silk or IB. For each experiment, a growth medium without nanoparticles was used as a reference.

#### 2.7.4. Cytotoxicity Assays

The cytotoxic effects of IB-SFNs and FA-IB-SFNs on HeLa, BT-474, SKBR3, and EA.hy926 cells were tested using the PrestoBlue Cell Viability Assay Reagent (Thermo Fisher Scientific, Waltham, MA, USA). Cells were seeded in 96-well plates at a concentration of 5 × 10^3^ cells/well. Twenty-four hours later, the cells were fed with fresh medium that contained different concentrations of nanoparticles. First, for the assays with HeLa cells, concentrations of nanoparticles between 3 × 10^−5^ and 1 mg/mL were used. Second, to compare the cytotoxic effect with the rest of the cell lines, a concentration of 3.125 × 10^−2^ mg/mL was used in all cases. The experiment involved the use of a control growth medium that did not contain nanoparticles. After 48 h, a PrestoBlue assay was conducted according to the manufacturer’s protocol. Fluorescence was measured using a microplate reader FLUOstar Omega (BMG LABTECH GmbH, Freiburg, Germany) spectrophotometer with an excitation wavelength of 530 nm and an emission wavelength of 590 nm. Each sample was tested in at least three independent sets.

#### 2.7.5. Nanoparticle Cellular Uptake

Cellular uptake of HeLa, BT-474, SKBR3, and EA.hy926 was determined for FITC-labeled SFNs, FITC-labeled IB-SFNs, and FITC-labeled FA-IB-SFNs. For these assessments, 2 × 10^5^ cells/well were seeded into a 12-well plate and incubated for 24 h. The culture medium was replaced by a fresh medium with 3.125 × 10^−2^ mg/mL of nanoparticles. After three washes with PBS, the cells were digested with trypsin to obtain cell suspensions. Cell-associated fluorescence was quantified by a Becton–Dickinson FACScalibur flow cytometer.

#### 2.7.6. Confocal Laser Scanning Microscopy

To confirm cellular uptake of nanoparticles, confocal laser scanning microscopy imaging was performed on HeLa and EA.hy926. Six hours after SFNs, IB-SFNs, and FA-IB-SFNs exposures, cells were washed with PBS 1x and fixed for 10 min in 4% paraformaldehyde. Actin filaments were stained for 30 min of incubation with rhodamine-phalloidin (Cytoskeleton, Inc., Denver, CO, USA) and DAPI (4′,6-diamidino-2-fenilindol) (Sigma-Aldrich, St. Louis, MO, USA). Confocal images were obtained with a Leica STELLARIS 8 inverted confocal laser scanning microscope, a white laser, and a FRET-FLIM module.

#### 2.7.7. Cell Cycle Arrest Assay

Studies of the effect of IB-SFNs and FA-IB-SFNs on cell cycle arrest were performed on HeLa, BT-474, SKBR3, and EA.hy926 cell lines. A total of 2 × 10^5^ cells/well were seeded in 12-well plates and allowed to fix to the plates for 24 h at 37 °C in a 5% CO_2_ and 95% humidity atmosphere. Then, IB-SFNs and FA-IB-SFNs at 3.125 × 10^2^ mg/mL were incubated for 48 h. Untreated cells were used as the control group. Cells were collected and centrifuged at 250× *g* for 10 min. After incubation, we collected and centrifuged the cells at 250× *g* for 10 min, washed them with phosphate-buffered saline (PBS), and suspended them in 200 μL of PBS for further analysis. Subsequently, a PBS (30%) and ethanol (70%) mixed solution was added to the cells and kept on ice for 30 min. The ethanol was then eliminated via centrifugation, and the cells were suspended in 400 µL of PBS, to which 50 µL of RNase solution and 50 µL of propidium iodide (PI) were added at a final concentration of 0.1 mg/mL and 40 mg/mL, respectively. Following this, cells were incubated in the dark for a period of 30 min. The PI fluorescence was measured for each cell in a Becton-Dickinson FACScalibur flow cytometer. In each case, 20,000 events were acquired.

#### 2.7.8. Apoptosis Assay

To analyze the effects of IB-SFNs and FA-IB-SFNs on cell death, experiments on apoptosis were completed. A total of 2 × 10^5^ cells were typically seeded in a 12-well plate for a period of 24 h. Afterwards, HeLa, BT-474, SKBR3, and EA.hy926 cells, along with IB-SFNs, FA-IB-SFNs, and their corresponding controls, were incubated. An apoptosis positive control with 8 µM camptothecin was also employed. After 24 h, cells were collected and washed twice with PBS as described above (no PBS-ethanol mixture was used in this case except for a necrosis positive control). Subsequently, 40 µL of a solution including Annexin V and PI (Annexin V-Fluos from Roche) and 1 mL of buffer (HEPES 10 nM, NaCl 140 mM, CaCl_2_ 5 mM, pH = 7.4) were added to the cell pellet. Cells were suspended in this solution and left in the dark at room temperature for 15 min. After, 200 µL of PBS was immediately included before the measurements. This experiment was carried out using a Becton–Dickinson FACScalibur flow cytometer, registering the emission at wavelengths of 620 and 525 nm for PI and Annexin V, respectively. In each case, 10,000 events were acquired.

## 3. Results and Discussion

### 3.1. Inverted Fluorescence Microscopy

**Figure 7:**
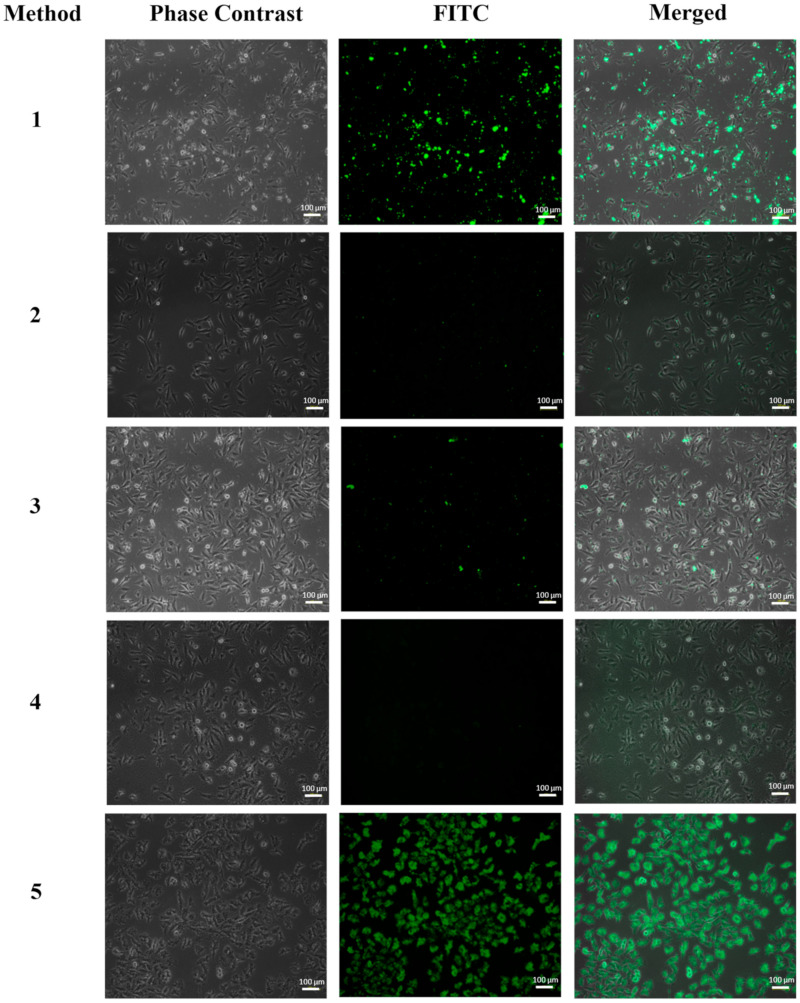
Fluorescence inverted microscopy analysis of the HeLa cell line at 6 h of exposure with FA-FITC-SFNs: Method 1, Method 2, Method 3, Method 4, and Method 5.

**Figure 8:**
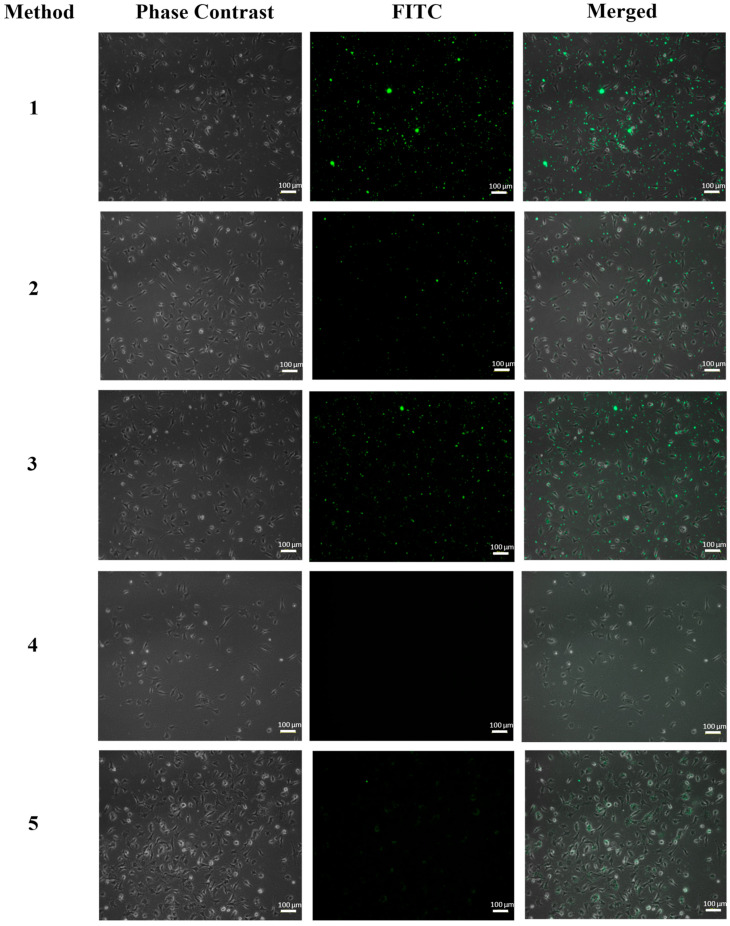
Fluorescence inverted microscopy analysis of the EA.hy926 cell line at 6 h of exposure with FA-FITC-SFNs: Method 1, Method 2, Method 3, Method 4, and Method 5.

To determine the best of the different functionalization methods described above, a simple assay by inverted fluorescence microscopy was performed on HeLa and EA.hy926 cells after 6 h of exposure to FA-FITC-SFNs, and the images obtained are shown in Figure 7 and Figure 8, respectively. Cells without nanoparticles were used as negative controls for both cell lines.

For the HeLa cell line (Figure 7), accumulation of FA-FITC-SFNs synthesized by Method 1 was observed, but the particles were not found in the cell cytoplasm, but were distributed throughout the culture medium. For the nanoparticles synthesized with Methods 2, 3, and 4, the microscopy images showed that the particles did not accumulate in the cells. However, for the nanoparticles synthesized by Method 5, the uptake of the FA-FITC-SFNs into the cytoplasm was significant.

In the case of the EA.hy926 cell line (Figure 8), the fluorescence spots of FA-FITC-SFNs synthesized by Methods 1, 2, and 3 were not positioned close to or along the cell morphology, which means that the cells had not internalized the nanoparticles. For those synthesized by Method 4, no fluorescence was even observed due to the dispersion of the nanoparticles in the medium. Finally, the FA-FITC-SFNs that were synthesized by Method 5 accumulate slightly inside the cells, however, at a very low concentration based on the fluorescence intensity. Therefore, it was concluded that Method 5 was the most effective for the purpose of targeting the nanoparticles to cancer cells (HeLa) overexpressing the folate receptor since, as discussed above, for healthy cells (EA.hy926) the fluorescence was much lower.

**Figure 9:**
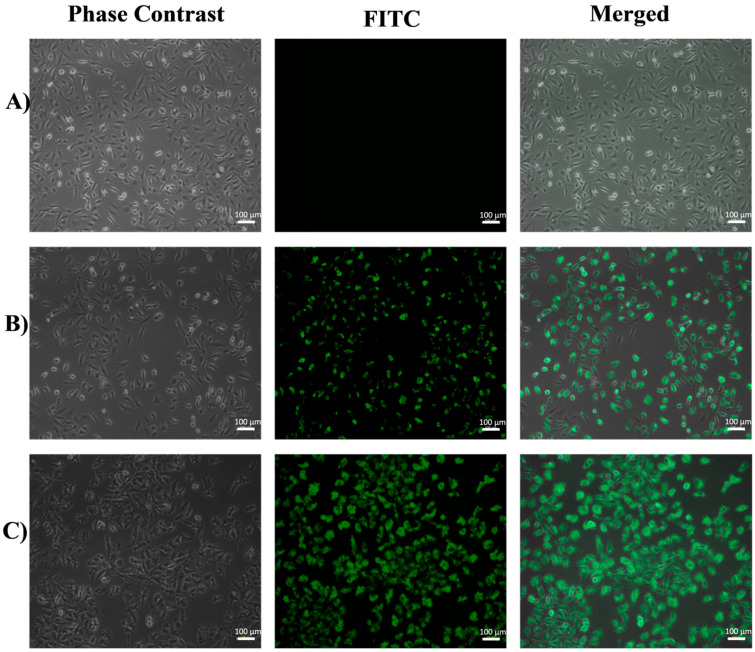
Fluorescence inverted microscopy analysis of the HeLa cell line exposed for 6 h to (**A**) no treatment (control), (**B**) FITC-SFNs, and (**C**) FA-FITC-SFNs.

**Figure 10:**
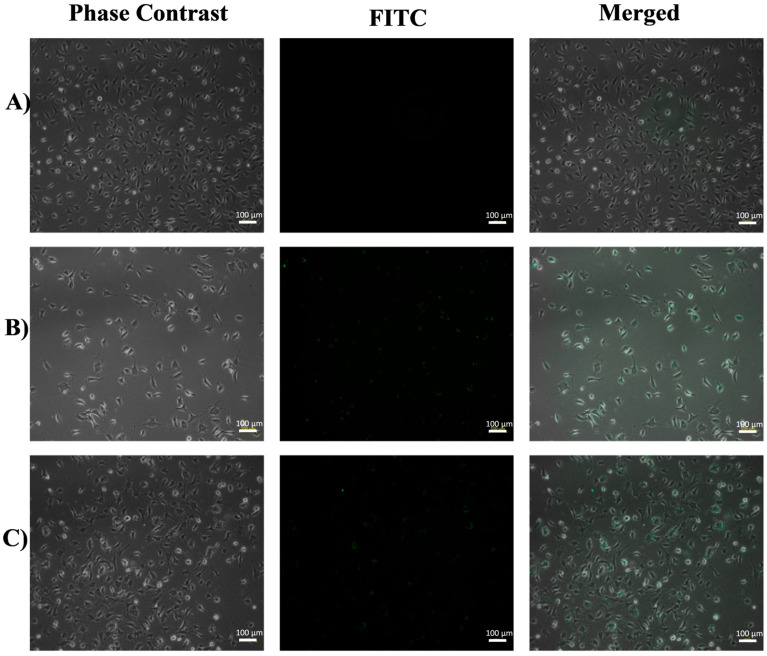
Fluorescence inverted microscopy analysis of the EA.hy926 cell line exposed for 6 h to (**A**) no treatment (control), (**B**) FITC-SFNs, and (**C**) FA-FITC-SFNs.

In order to study whether the FA-FITC-SFNs synthesized by Method 5 were optimal for cell targeting, they were tested on both cell lines at 3 and 6 h of exposure, and comparisons with FITC-SFNs were carried out. For the HeLa cell line (carcinogenic), after 3 h of exposure to FITC-SFNs (Figure S2 of Supporting Information), an uptake was observed, but it was more evident when exposed to FA-FITC-SFNs Figure S2C of Supporting Information) based on the spots corresponding to the FA-FITC-SFNs that accumulated inside the cells while maintaining cell shape. At 6 h of exposure, more entry (higher fluorescence intensity) was observed with the FITC-SFNs (Figure 9B), and this effect was even more pronounced for the FA-FITC-SFNs (Figure 9C). The results for the EA.hy926 cell line (healthy cells) at 3 h (Figure S3 of Supporting Information) and 6 h (Figure 10) of exposure revealed a very low increase in uptake of all the nanoparticles, demonstrating the effectiveness of Method 5 for the purpose of this work. For these reasons, functionalized nanoparticles prepared by Method 5 were chosen for full characterization.

### 3.2. Hydrodynamic Mean Diameter and Z-Potential of the Nanoparticles

**Figure 11:**
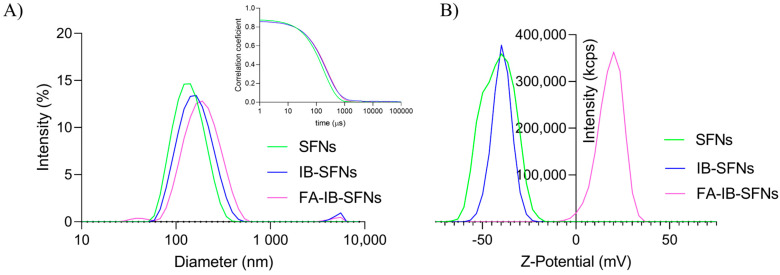
(**A**) Size distribution based on the intensity of SFNs, IB-SFNs, and FA-IB-SFNs with an insert of the correlation coefficient for the three samples; and (**B**) Z- potential distributions of SFNs, IB-SFNs, and FA-IB-SFNs.

Figure 11A shows the size distribution of SFNs, IB-SFNs, and FA-IB-SFNs (Method 5). The three systems present narrow monomodal distributions with polydispersity indices (PdI) of 0.124, 0.213, and 0.220, respectively. The hydrodynamic mean diameter (expressed as Z-average) and diffusion coefficients for SFNs were 129 nm and 3.83 µ^2^/s, respectively. The IB-SFNs presented values of 156.1 nm and 3.15 µ^2^/s, respectively, while the FA-IB-SFNs values were 203.5 nm and 2.94 µ^2^/s, respectively. A slight augment of the Z-average values was detected when the drug and the FA were incorporated into the SFNs, but it can be concluded that the sizes were suitable. The raw correlograms from which the size distribution by intensity and Z-average were determined can be seen in the insert of Figure 11A. For Z-potential and electrophoretic mobility, SFNs showed a value of −41.3 mV and −3.234 µmcm/Vs, respectively; the IB-SFNs presented a value of −36 mV and −2.822 µmcm/Vs, respectively; and the FA-IB-SFNs presented values of 21.6 mV and 0.0124 µmcm/Vs, respectively. Figure 11 shows the Z-potential distributions for the three systems. The positive charge of FA was the cause of the positive value of the Z-potential of FA-IB-SFNs. However, the value was sufficiently high for adequate stability in water suspensions.

### 3.3. Field Emission Scanning Electron Microscopy (FESEM)

**Figure 12:**
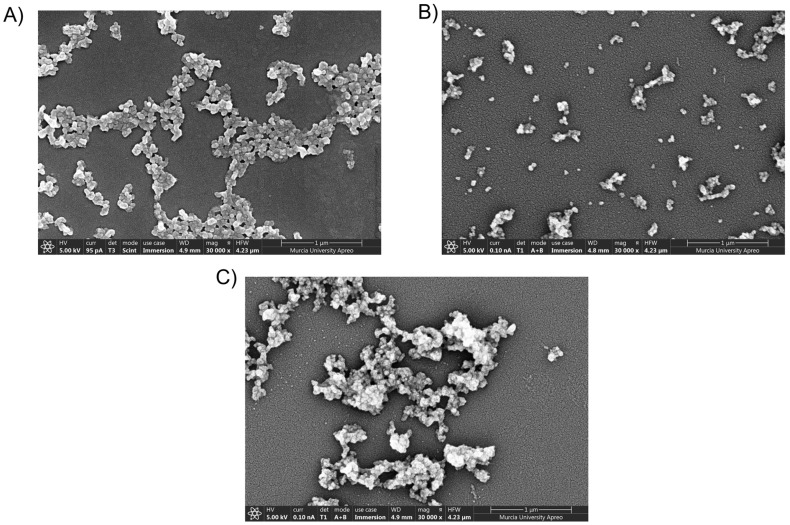
Field emission scanning electron microscopy (30,000×) images of (**A**) SFNs, (**B**) IB-SFNs, and (**C**) FA-IB-SFNs.

The size and morphology of SFNs, IB-SFNs, and FA-IB-SFNs (Method 5) were examined by FESEM (Figure 12). The nanoparticles were globular, granular, and quasispheroidal with a size of around 100 nm, as observed for other drug-loaded SFNs [14]. Homogeneous size distribution can be observed, and the diameters were smaller than those obtained by DLS. In the case of DLS, particles are dispersed in water, whereas in FESEM, the particles are dried. Therefore, swelling from the DLS effect would account for this difference.

### 3.4. Drug Loading Content (DLC) and Encapsulation Efficiency (EE)

According to a previous method [42], from the corrected and normalized ATR-FTIR spectra of IB and SFN, a calibration curve was constructed, and a good linear correlation between IB concentration and absorbance was found (Figure S4 and Table S1 of Supporting Information). After the IB loading experiments, three independent samples of IB-SFNs were analyzed to determine their DLC values by ATR-FTIR. The average DLC for IB was 10.2 ± 0.43 % and 26.67% for EE.

### 3.5. In Vitro Cytotoxicity

**Figure 13:**
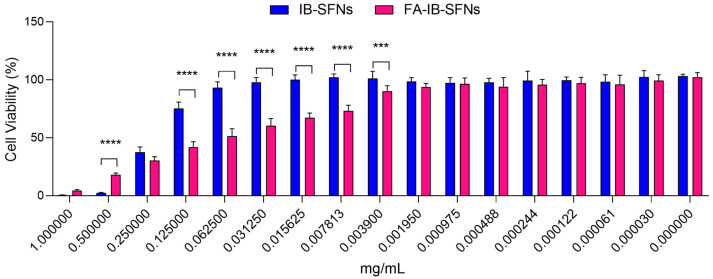
Cytotoxic effect of FA-IB-SFNs and IB-SFNs on the HeLa cell line. Data are expressed as a percentage of cell viability ± SD vs. concentration. *** indicates *p* < 0.001 and **** indicates *p* < 0.0001, comparing IB-SFNs with FA-IB-SFNs. Untreated cells were used as a control.

The cytotoxic effect of IB-SFNs and FA-IB-SFNs (Method 5) on HeLa cells was evaluated. This cell line was selected according to its origin and high-level expression of folate receptor [43]. Cervical carcinoma-derived HeLa cells have frequently been employed in cytotoxicity experiments. The cells were exposed to a variety of intensities of IB-SFNs and FA-IB-SFNs (3 × 10^−5^–1 mg/mL) for 48 h, and then the cell viability was assessed (Figure 13). FA-IB-SFNs were more cytotoxic on HeLa cells than IB-SFNs in the concentration range of 0.00195 to 0.25 mg/mL. For lower concentrations than 0.00195 mg/mL, cell viability was approximately 100% for both treatments. At concentrations higher than 0.25 mg/mL, both treatments killed the cells. The results supported working with lower concentrations of FA-IB-SFNs, which yielded a higher reduction of cell viability, which presumably could enhance the treatments with IB.

**Figure 14:**
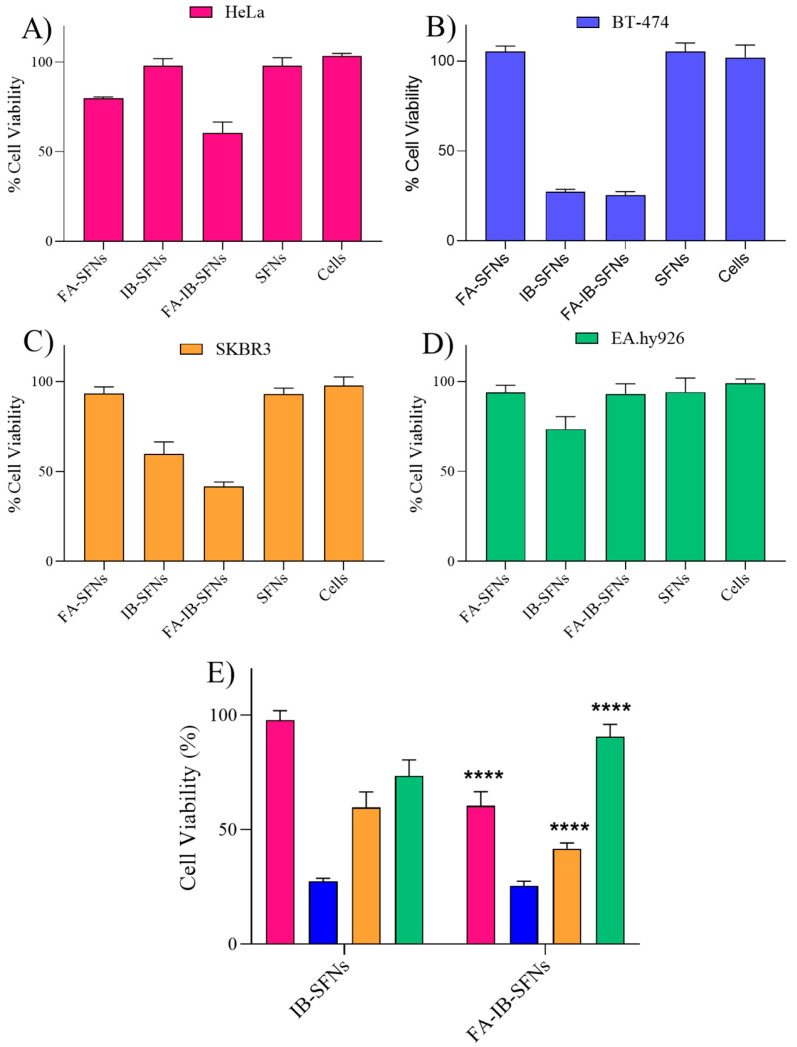
Cytotoxic effect of SFNs, FA-SFNs, IB-SFNs, and FA-IB-SFNs on (**A**) HeLa, (**B**) BT-474, (**C**) SKBR3, and (**D**) EA.hy926 cell lines at 0.03125 mg/mL. (**E**) Cytotoxic effect comparison between IB-SFNs and FA-IB-SFNs on HeLa, BT-474, SKBR3 and EA.hy926 cell lines at 0.03125 mg/mL. Data are expressed as a percentage of cell viability ± SD vs. concentration. **** indicates *p* < 0.0001, comparing IB-SFNs vs. FA-IB-SFNs with each cell line.

Figure 14 shows the comparison of the cytotoxic effect of SFNs, FA-SFNs, IB-SFNs, and FA-IB-SFNs at 0.03125 mg/mL on the four cell lines studied (HeLa, SKBR3, BT-474, and EA.hy926). SFNs and FA-SFNs were not significantly cytotoxic to any of the cell lines, as expected. Comparing IB-SFNs and FA-IB-SFNs, FA-IB-SFNs were more cytotoxic on HeLa and SKBR3 due to the presence of the FA (Figure 14E). This corroborates the targeting of FA on the tumor cells. However, in the case of BT-474 cells, this effect was slight, with about the same cell viability of around 25% as obtained for IB-SFNs compared to FA-IB-SFNs. This may be due to BT-474 cells being more sensitive to the drug IB; therefore, at this concentration, cell viability was significantly reduced. In contrast, the healthy EA.hy926 cells were less affected by the FA-IB-SFNs than by IB-SFNs, which constitutes a good starting point for future studies considering the targeting effect of FA in SFNs.

### 3.6. In Vitro Cellular Uptake by Flow Cytometry

**Figure 15:**
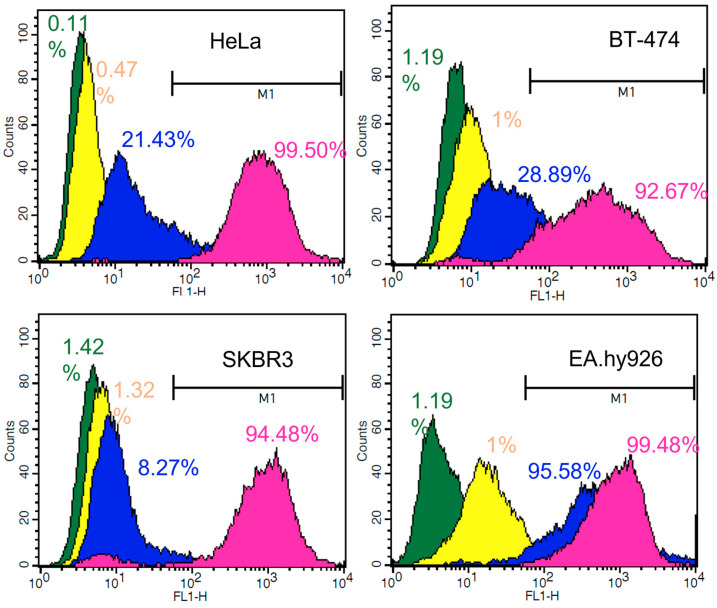
Detection and quantification by flow cytometry of HeLa, BT-474, SKBR3, and EA.hy926 and BT-474 cellular uptake of FITC-labeled SFNs (yellow), FITC-labeled IB-SFNs (blue), and FITC-labeled FA-IB-SFNs (pink) at a concentration of 0.01325 mg/mL after 24 h of cell exposure. In all cases, cells without any treatment were used as the negative control (green).

In order to evaluate the increase in the entry of functionalized nanoparticles into cells (FA-IB-SFNs) by Method 5 compared to non-functionalized particles (IB-SFNs), uptake was quantified using flow cytometry (Figure 15). The median intensity of the fluorescence (FITC) signal in the populations of HeLa, BT-474, SKBR3, and EA.hy926 was studied using flow cytometry. After culture, the cells were exposed to 0.03125 mg/mL of FITC-SFNs, FITC-IB-SFNs, and FITC-FA-IB-SFNs, and the median cell fluorescence intensity of FITC-labeled SFNs was close to the controls for all cell lines. The fluorescence intensities of FITC-IB-SFNs and FITC-FA-IB-SFNs increased relative to controls, implying that both varieties of FITC-labeled nanoparticles were internalized by cells (Figure 15). The results showed that the fluorescence intensity was less when cells were exposed to FA-free nanoparticles (IB-SFNs) when compared to FA functionalized nanoparticles (FA-IB-SFNs), showing maxima at 8.27% and 94.48%, respectively, in SKBR3 cells; at 21.43% and 99.50%, respectively, in HeLa cells; at 28.89% and 92.67%, respectively, in BT-474 cells; and at 95.58% and 99.48%, respectively, in EA.hy926 cells. These results confirm the increased internalization of functionalized nanoparticles into cancer cells via folate receptors. However, this increase is not reflected in healthy cells when exposed to functionalized nanoparticles.

### 3.7. Confocal Laser Scanning Microscopy

**Figure 16:**
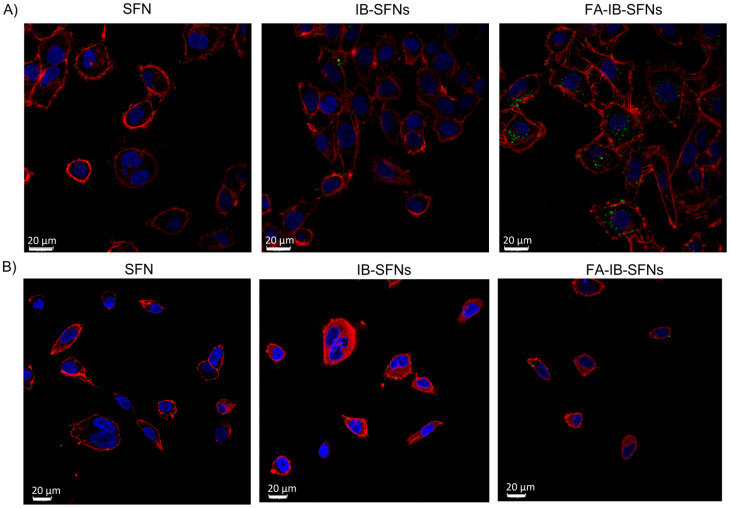
Confocal laser scanning microscopy of (**A**) HeLa and (**B**) EA.hy926 cell lines after 6 h of exposure to FITC-labeled SFNs, FITC-labeled IB-SFNs, and FITC-labeled FA-IB-SFNs. Cytoplasmic actin filaments were stained with phalloidin (red) and nuclei with DAPI (blue).

To test the potential of FA-IB-SFNs as possible carriers of pharmacological molecules, the cellular localization of FITC-IB-SFNs and FA-IB-SFNs to HeLa and EA.hy926 cells was examined. These cell lines were chosen as representatives of cancerous and healthy tissues, respectively. In the case of HeLa cells, they have been widely used in folate-receptor studies [43], and EA.hy926 are healthy cells that coat the inside of blood vessels and are one of the best characterized human vascular endothelial cell lines [44]. In both cases, cellular uptake of FITC-labeled nanoparticles was observed by confocal laser scanning microscopy (Figure 16).

FITC-SFNs and FITC-IB-SFNs did not show any significant entrance on tumor or healthy cells after 6 h (Figure 16). However, in the case of FITC-FA-IB-SFNs, significant cell uptake was observed for the HeLa tumor cells but not for the EA.hy926 healthy cells. These results corroborate the targeting role of FA in SFNs.

### 3.8. Influence of FA-IB-SFN and IB-SFN Treatments on the Cell Cycle

**Figure 17:**
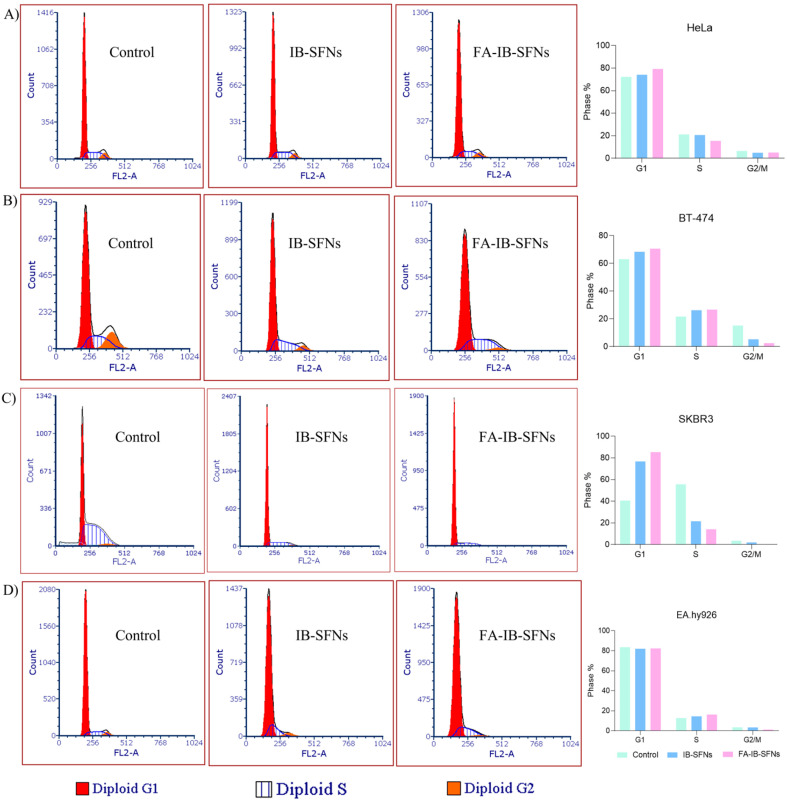
Cell cycle of (**A**) HeLa, (**B**) BT-474, (**C**) SKBR3, and (**D**) EA.hy926 cells exposed to 0.03125 mg/mL of IB-SFNs and FA-IB-SFNs. Untreated cells were used as a control.

To study the inhibition of cell growth through cell cycle arrest, the effect of IB-SFNs and FA-IB-SFNs at 0.03125 mg/mL were examined on HeLa, BT474, SKBR3, and EA.hy926 cells for 48 h by flow cytometry and compared to the control (Figure 17A). In HeLa cells, the percentage of cells in all phases remained approximately constant with respect to the control for IB-SFNs treatment, ranging from 72.25% to 74.24% in the G1 phase, from 21.22% to 20.68% in the S phase, and from 6.53% to 5.08% in the G2/M phase, respectively. However, when HeLa cells were exposed to FA-IB-SFNs, G1 phase increased to 79.27%, S phase decreased to 15.52%, and G2/M phase decreased to 5.22%, suggesting the HeLa cells became arrested in G1 phase due to the functionalized nanoparticle treatment. In the BT-474 cells (Figure 17B), the number of cells in the G1 phase increased with respect to the control for IB-SFNs and FA-IB-SFNs from 63.12% to 68.4% and 70.72%, respectively. In the case of S phase, the cell number decreased in all cases from 21.68% (control) to 26.31% (IB-SFNs) and 26.73% (FA-IB-SFNs). The G2/M phase cell number significantly decreased from 15.2% to 5.29% with IB-SFNs and to 2.55% with FA-IB-SFNs. In light of these results, it was concluded that FA-IB-SFNs inhibited the cell growth of BT-474 cells due to the arrest of the cells in the G1 phase to a greater extent than IB-SFNs. In Figure 17C, the numbers in G1 phase in SKBR3 cells increased with respect to the control when cells were treated with IB-SFNs, from 40.71% to 76.80%, and significantly increased to 85.39% when cells were exposed to FA-IB-SFNs. The S cell number significantly decreased from 55.70% to 21.50% with IB-SFNs and to 14.22% with FA-IB-SFNs. The number of cells in the G2/M phase also decreased from 3.61% to 2.05% when cells were treated with IB-SFNs and to 0.39% when treated with FA-IB-SFNs. The effect of the drug IB on the SKBR3 cell cycle was more evident when the drug was incorporated into functionalized nanoparticles, suggesting that FA-IB-SFNs cause SKBR3 cells to become arrested in the G1 phase and then undergo apoptosis rather than proceed to the S phase. Ibrutinib treatment in HER2-overexpressing breast cancer cell lines (SKBR and BT-474) caused growth inhibition by arresting cells in the G1 phase of the cell cycle. The healthy cells EA.hy926 are shown in Figure 17D [45]. In the G1 phase, the number of cells for the different treatments remained constant compared to the control, ranging from 83.61% to 82% (IB-SFNs) and to 82.42% (FA-IB-SFNs). In the S phase, a small increase was noted compared to the control, from 12.81% to 14.53% (IB-SFNs) and to 16.31% (FA-IB-SFNs). Finally, in the G2/M phase, the number of cells decreased when treated with functionalized nanoparticles compared to controls, from 3.57% to 1.27%. When EA.hy926 were treated with IB-SFNs, the percentage of cells remained around 3.57%, similar to the control.

When treated with FA-IB-SFNs, the cell cycle was arrested in the G1 phase for all cancer lines studied, but was arrested to a lesser extent when treated with IB-SFNs. These results suggest that folic acid accelerates this process. Keeping the cells to a greater extent in the G1 phase of the cell cycle is interesting because the cells cannot continue to the S and G2/M phases, so they cannot synthesize DNA or undergo mitosis to continue proliferating. The same outcome happened with healthy cells, but to a lesser extent. Although the cell cycle can be modified to some extent after 24 h of exposure to the nanoparticles, treatment with folic acid at 48 h would not kill healthy cells but would kill the cancer cells, as can be seen from the cytotoxicity results earlier. Several in vitro studies with IB found in the literature showed the ability to inhibit cell proliferation and induce cell cycle arrest in various cancer cells [33,46].

### 3.9. Apoptosis Assays

**Figure 18:**
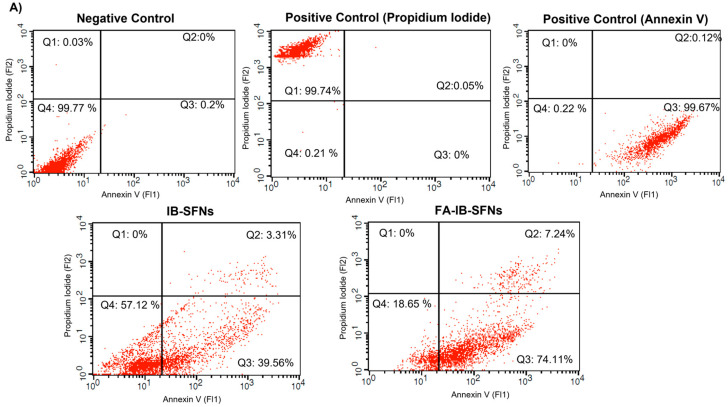

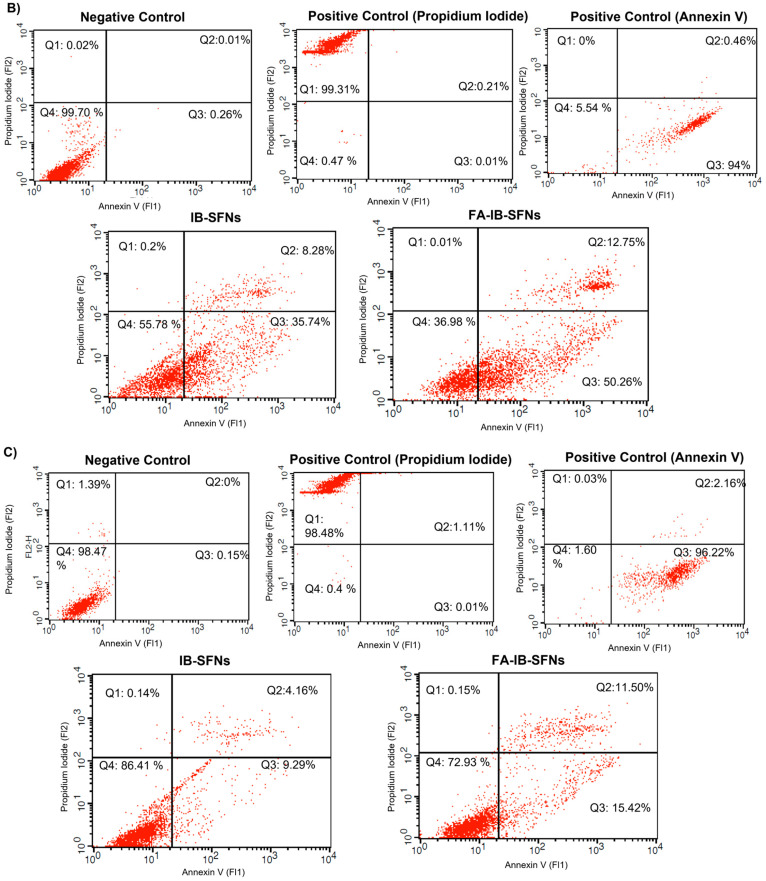

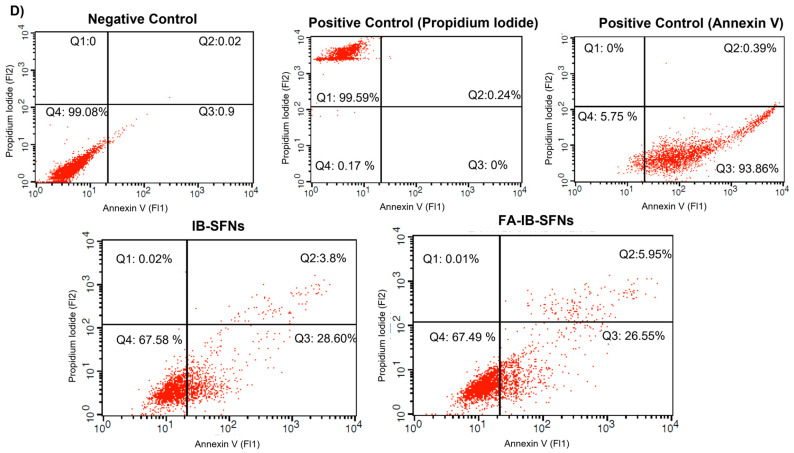
Flow cytometry analysis of the apoptosis rate in (**A**) HeLa, (**B**) BT-474, (**C**) SKBR3, and (**D**) EA.hy926 cells after 24 h of treatment with IB-SFNs and FA-IB-SFNs. As a negative control, cells not subjected to treatment were taken into consideration. Camptotecin was used as an apoptosis positive control (annexin V) and a necrosis positive control (propidium iodide), which were established by assessing cells that had not been treated, fixed, or stained.

To make an assessment of cell death due to the consequence of IB-SFNs and FA-IB-SFNs on HeLa, BT474, SKBR3, and EA.hy926 cells, the Annexin-V-Fluos Apoptosis Detection Kit was applied to measure the apoptosis caused by 24 h of exposure. The Annexin-V-Fluos stained graph is composed of four quadrants (Q), where Q1 signifies necrotic cells, Q2 and Q3 denote late and early apoptotic cells, respectively, and Q4 are the living cells. Untreated control live cells were used, and the positive apoptosis control involved cells exposed to camptotecin, a chemical compound that prompts cellular apoptosis, and the positive control for necrosis was fixed cells stained with propidium iodide. In Figure 18A, an increase in the apoptotic effect (Q2 + Q3) was observed in the HeLa cells after treatment with FA-IB-SFNs (81.35%) compared to IB-SFNs (42.87%). When treated with IB-SFNs, 44.02% of the BT-474 cells analyzed were in an apoptotic phase, whereas with treatment with FA-IB-SFNs, an increase to 83% was observed (Figure 18B). In SKBR3 cells (Figure 18C), the treatment with IB-SFNs resulted in 13.45% of the cells being in the apoptotic phase, whereas an increase of the apoptotic phase was observed when treated with FA-IB-SFNs to 26.92%. Finally, the number of EA.hy926 cells in the apoptotic phase remained constant when treated with IB-SFNs and FA-IB-SFNs, 32.4% and 32.5%, respectively. In contrast, the percentage of cells that were dead due to necrosis was negligible for all cell lines and all treatments.

In light of the results of the apoptosis analysis, we can conclude that FA-IB-SFNs induced apoptosis in all cell lines, leading to more significant antiproliferative effects on all cancer cell lines because of the increased number of cells in the apoptotic phase (higher than in healthy cells, EA.hy926). These results suggest that FA-IB-SFNs inhibit cell proliferation in cancer cell lines by inducing apoptosis to a greater extent than IB-SFNs. Previous studies have also found that ibrutinib induces apoptosis in HER2 overexpressing breast cancer cell lines [46]. These results also corroborate previous findings that the presence of FA in the nanoparticles enhances the therapeutic efficiency of the drug IB on tumor cells by inducing apoptosis. In the case of healthy cells, significant differences between the treatments with IB-SFNs or FA-IB-SFNs were not found.

## 4. Conclusions

The synthesis and characterization of the functionalized nanoparticles FA-IB-SFNs are described, with the aim of improving the uptake and antitumor activity of IB. The optimal method for functionalizing the nanoparticles was chosen as Method 5. The hydrodynamic diameter found for the FA-IB-SFNs was 203.5 nm, with a narrow size distribution and Z-potential of 21.6 mV. In addition, a DLC value of 10.2% and an EE of 26.67% were obtained. An enhanced degree of growth inhibition of FA-IB-SFNs (compared to IB-SFNs) against HeLa, BT-474, and SKBR3 cancer cell lines was observed. EA.hy926 healthy cell line viability was almost unmodified by treatment with functionalized nanoparticles. The relative non-toxicity of the unloaded SFNs and FA-SFNs indicated good cytocompatibility of the nanocarrier, and the cytotoxic effect was caused by the drug. The cellular uptake studies confirmed high internalization of FA-IB-SFNs into cancer cells after 24 h compared to IB-SFNs, suggesting that cellular uptake was folate-receptor mediated, which is overexpressed in cancer cells. FA-IB-SFNs induced cell arrest in the G1 phase and increased apoptosis in all cancer cells compared to IB-SFNs. The mode of cell death post-FA-IB-SFNs was analyzed by flow cytometry and confocal laser scanning microscopy, and the results suggested that apoptosis was the primary mode of cancer cell death induced by FA-IB-SFNs. These results indicated that the nanocarrier developed can be used for drug targeting applications by enhancing the uptake of the IB in cancer cells with overexpressed folate receptors. In vivo studies would be required to corroborate these in vitro findings.

## Data Availability

Not applicable.

## Supplementary Materials

The following supporting information can be downloaded at: https://www.mdpi.com/article/10.3390/pharmaceutics15041186/s1. Figure S1. Ibrutinib; orally bioavailable, irreversible inhibitor of Bruton’s Tyrosine Kinase; molecular formula C_25_H_24_N_6_O_2_; chemical name 1-[(3R)-3-[4-amino-3-(4-phenoxyphenyl)pyrazolo[3,4-d]pyrimidin-1-yl]piperidin-1-yl]prop-2-en-1-one; molecular weight-440.50 Da [38]. Figure S2. Fluorescence inverted microscopy analysis of HeLa cell line exposed for 3 h to (A) Cells with no treatment (Control), (B) FITC-SFNs and (C) FA-FITC-SFNs. Figure. S3. Fluorescence inverted microscopy analysis of EA.hy926 cell line exposed for 3h to (A) Cells with no treatment (Control), (B) FITC-SFNs and (C) FA-FITC-SFNs. Figure S4. (A) Absorbance spectra of calibration samples; (B) Calibration curve obtained from seven samples measured in triplicate. Some error bars not visible due to the low SD. Table S1. Calibration curve parameters.
