# Supplementary material for: Folic Acid-Modified Ibrutinib-Loaded Silk Fibroin Nanoparticles for Cancer Cell Therapy with Over-Expressed Folate Receptor
Source: Pharmaceutics. 2023 Apr 7;15(4):1186. doi: 10.3390/pharmaceutics15041186 (PMC10146313; doi:10.3390/pharmaceutics15041186)
Supplement: Supplementary file 1 [file pharmaceutics-15-01186-s001.zip › pharmaceutics-2296238-supplementary.pdf]

pharmaceutics

Supplementary Materials: Folic acid-modified Ibrutinib-loaded silk fibroin nanoparticles for cancer cell therapy with over-expressed folate receptor

Marta G. Fuster, Mercedes G. Montalbán, Imane Moulefera , Gloria Víllora and David L. Kaplan

Supplementary Information:

IBRUTINIB

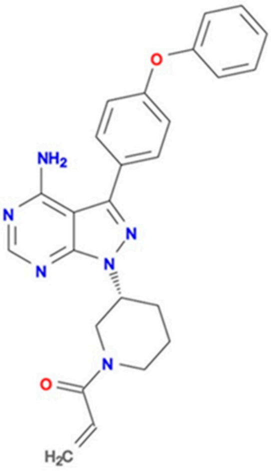

Figure S1. Ibrutinib; orally bioavailable, irreversible inhibitor of Bruton's Tyrosine Kinase; molecular formula $C_{25}H_{24}N_6O_2$; chemical name 1-[(3R)-3-[4-amino-3-(4-phenoxyphenyl)pyrazolo[3,4-d]pyrimidin-1-yl]piperidin-1-yl]prop-2-en-1-one; molecular weight-440.50 Da [1].

INVERTED FLUORESCENCE MICROSCOPY

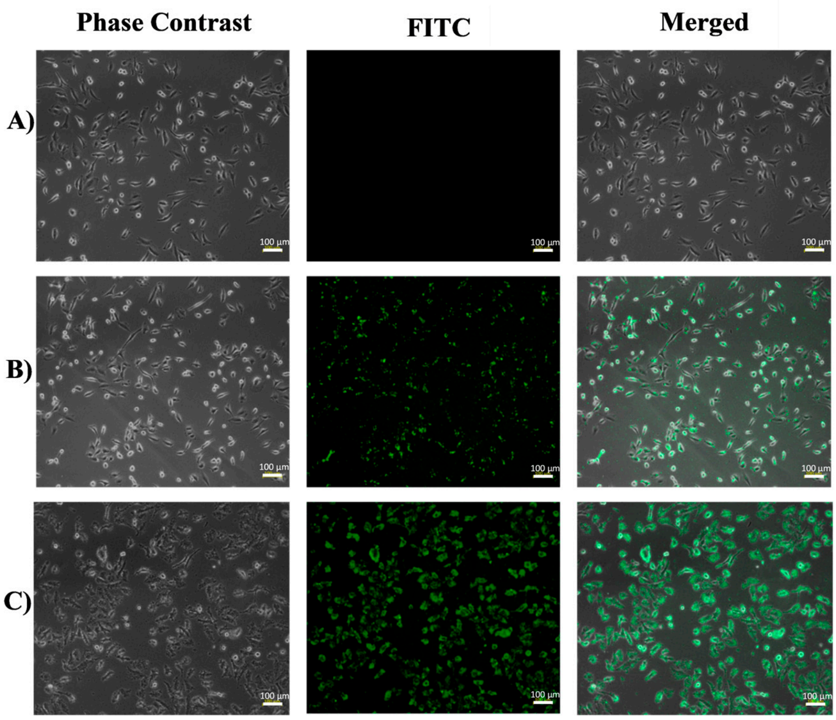

Figure S2. Fluorescence inverted microscopy analysis of HeLa cell line exposed for 3h to A) Cells with no treatment (Control), B) FITC-SFNs and C) FA-FITC-SFNs

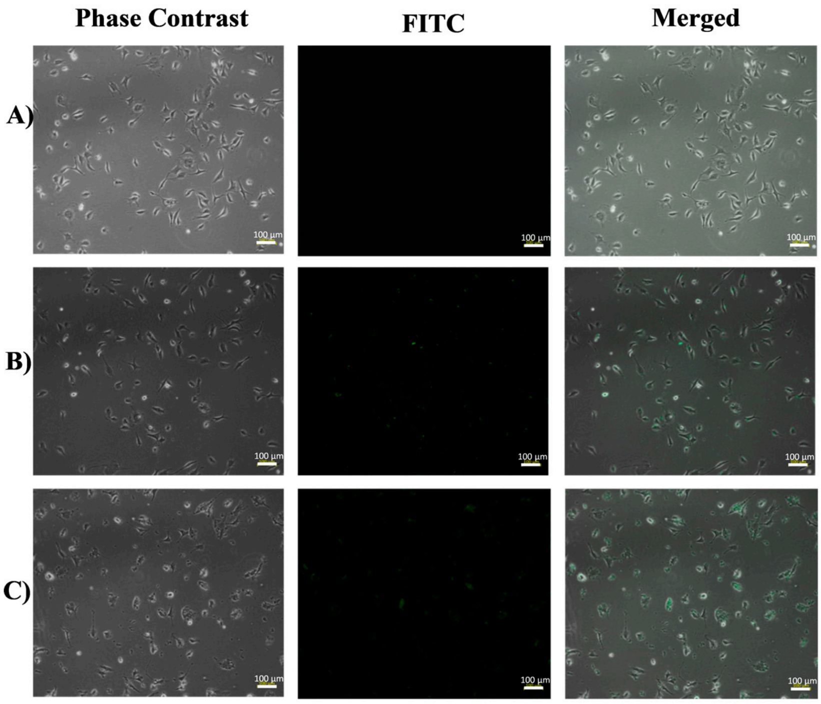

Figure S3. Fluorescence inverted microscopy analysis of EA.hy926 cell line exposed for 3h to A) Cells with no treatment (Control), B) FITC-SFNs and C) FA-FITC-SFNs.

DRUG LOADING CONTENT (DLC) AND ENCAPSULATION EFFICIENCY (EE)

Acording to a previously method [2], from the base line corrected and normalized ATR-FTIR spectra of IB and SFNs, the peak at 1103 cm-1, a signal from the drug, shows low-to-no interference with the polymeric matrix of the nanoparticles. From the peak at this wavenumber, a calibration curve was constructed from samples with a constant SFN mass and increasing IB mass, i.e. from 0 to 12.5% of IB/SFN (w/w) to generate a calibration curve (Figure S4 A). A good linnear correlation was found between the concentration of IB and the normalized absorbance, with an R2 of 0.98870 (Table S1). Finally, DLC, defined in Equation (S1), was obtained directly from the calibration line. EE, defined as Equation (S2), was calculated from DLC and the initial mass of IB in the loading solution by mass balance.

$$DLC\ (\%) = \frac{Mass\ of\ IB\ loaded\ onto\ SFNs}{Mass\ of\ IB{-}SFNs} \times 100 \quad \text{(S1)}$$

$$EE\ (\%) = \frac{Mass\ of\ IB\ loaded\ onto\ SFNs}{Mass\ of\ IB\ added\ to\ SFNs} \times 100 \quad \text{(S2)}$$

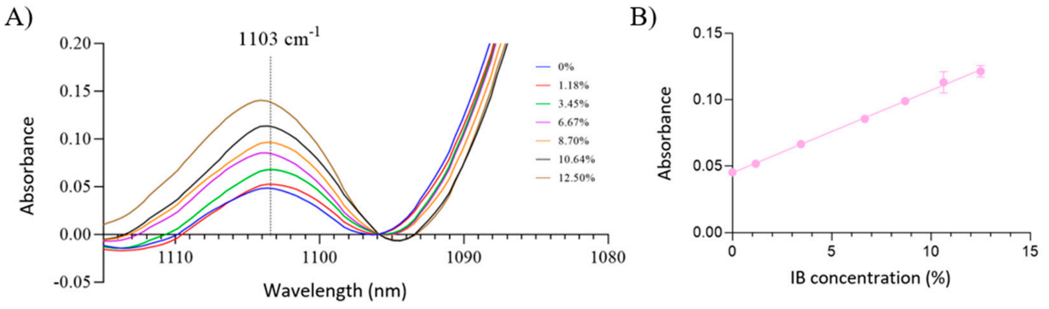

Figure S4. (A) Absorbance spectra of calibration samples; (B) Calibration curve obtained from seven samples measured in triplicate. Some error bars not visible due to the low SD.

Table S1. Calibration curve parameters.

	Best-Fit Values	95% Confidence Intervals
**Slope**	0.006203	0.005896 to 0.006511
**Y-intercept**	0.04499	0.04274 to 0.04725
**R^2**	0.98870	

After the IB loading experiments, three independent samples of IB-SFNs were analyzed to determine their DLC values by ATR-FTIR. The baseline was corrected, and the spectra were normalized, while the absorbance at 1103 cm-1 was interpolated in the calibration curve (Figure S2B).

References

1 Schwarzbich, M.A.; Witzens-Harig, M.. Ibrutinib. *Recent Results Cancer Res.* **2014**, *201*, 259–267. https://doi.org/10.1007/978-3-642-54490-3_17

2 Carissimi, G.; Montalbán, M.G.; Víllora, G.; Barth, A. Direct quantification of drug loading content in polymeric nanoparticles by infrared spectroscopy. *Pharmaceutics* **2020**, *12*, 912. https://doi.org/10.3390/pharmaceutics12100912

Disclaimer/Publisher's Note: The statements, opinions and data contained in all publications are solely those of the individual author(s) and contributor(s) and not of MDPI and/or the editor(s). MDPI and/or the editor(s) disclaim responsibility for any injury to people or property resulting from any ideas, methods, instructions or products referred to in the content.
